# A D-Shaped Bileaflet Bioprosthesis which Replicates Physiological Left Ventricular Flow Patterns

**DOI:** 10.1371/journal.pone.0156580

**Published:** 2016-06-03

**Authors:** Sean Guo-Dong Tan, Sangho Kim, Jimmy Kim Fatt Hon, Hwa Liang Leo

**Affiliations:** 1 Department of Biomedical Engineering, National University of Singapore, Faculty of Engineering, Block E4, #04–08, 4 Engineering Drive 3, Singapore 117583, Singapore; 2 Department of Surgery, National University of Singapore, Yong Loo Lin School of Medicine, Kent Ridge Road, Singapore 119228, Singapore; University at Buffalo, SUNY, UNITED STATES

## Abstract

Prior studies have shown that in a healthy heart, there exist a large asymmetric vortex structure that aids in establishing a steady flow field in the left ventricle. However, the implantation of existing artificial heart valves at the mitral position is found to have a negative effect on this physiological flow pattern. In light of this, a novel D-shaped bileaflet porcine bioprosthesis (GD valve) has been designed based on the native geometry mitral valve, with the hypothesis that biomimicry in valve design can restore physiological left ventricle flow patterns after valve implantation. An in-vitro experiment using two dimensional particle velocimetry imaging was carried out to determine the hemodynamic performance of the new bileaflet design and then compared to that of the well-established St. Jude Epic valve which functioned as a control in the experiment. Although both valves were found to have similar Reynolds shear stress and Turbulent Kinetic Energy levels, the novel D-shape valve was found to have lower turbulence intensity and greater mean kinetic energy conservation.

## Introduction

The performance of current bio-prosthesis designs have traditionally been evaluated on conventional parameters such as trans-vavular pressure drop, effective orifice area, para-valvular leakage and Reynolds shear stress levels[1]. Although the well-established tri-leaflet design has been clinically proven to have long term durability[2] and low levels of thromboembolism[3], recent studies have suggested that the implantation of an artificial valve at the mitral position can significantly alter left ventricle flow field[4–6], as various parameters including valve geometry and orientation can have an effect on hemodynamics[7].

In a healthy heart, the left ventricular flow field consists of an asymmetrical clockwise vortex structure that smoothly redirects the incoming blood from the mitral position to the left ventricular outflow tract and towards the aorta [4, 8, 9]. This physiological flow pattern minimizes kinetic energy loss due to turbulent fluctuation, conserving the energy provided by the incoming jet at peak flow[8]. Furthermore, it has been established that vortex formation in the left ventricle is an indicator of cardiac health[10, 11], therefore abnormal or disturbed flow patterns might predict impending cardiac diseases before any overt manifestation of symptoms [12, 13].

Thus, the conventional parameters used to determine hemodynamic performance for mitral valves are no longer sufficient without the consideration of the valve’s effects on left ventricular flow patterns. Future mitral valve designs need to take into account vortex formation in the left ventricle, by seeking to preserve the physiological asymmetric vortex structures[9], since it has been suggested that the D-shaped annulus in tandem with the anterior mitral leaflet might contribute to the formation of an asymmetrical vortex downstream[14]. In addition, an axisymmetric index derived from the impulse of a vortex formed, recently proposed by Kheradvar et. al.; can potentially be an additional parameter by which the hemodynamic performance of mitral valves can be quantified[15].

This study proposes a new D-shape bi-leaflet bio-prosthesis (GD Valve) that has its geometry and dimensions derived from the native human mitral valve, enabling the generation of large asymmetrical clockwise vortices in the left ventricle (LV) similar to that observed in a healthy heart, resulting in lower turbulence and a conservation of kinetic energy as the incoming jet is redirected smoothly towards the outflow tract (LVOT). In order to test this hypothesis, the in-vitro hemodynamic performance of the GD Valve is benchmarked against that of the well-established Epic Valve (St. Jude Medical) through comparison of flow field downstream of the valves inside the left ventricle.

## Materials and Methods

### St. Jude Medical Epic Valve

**Fig 1:**
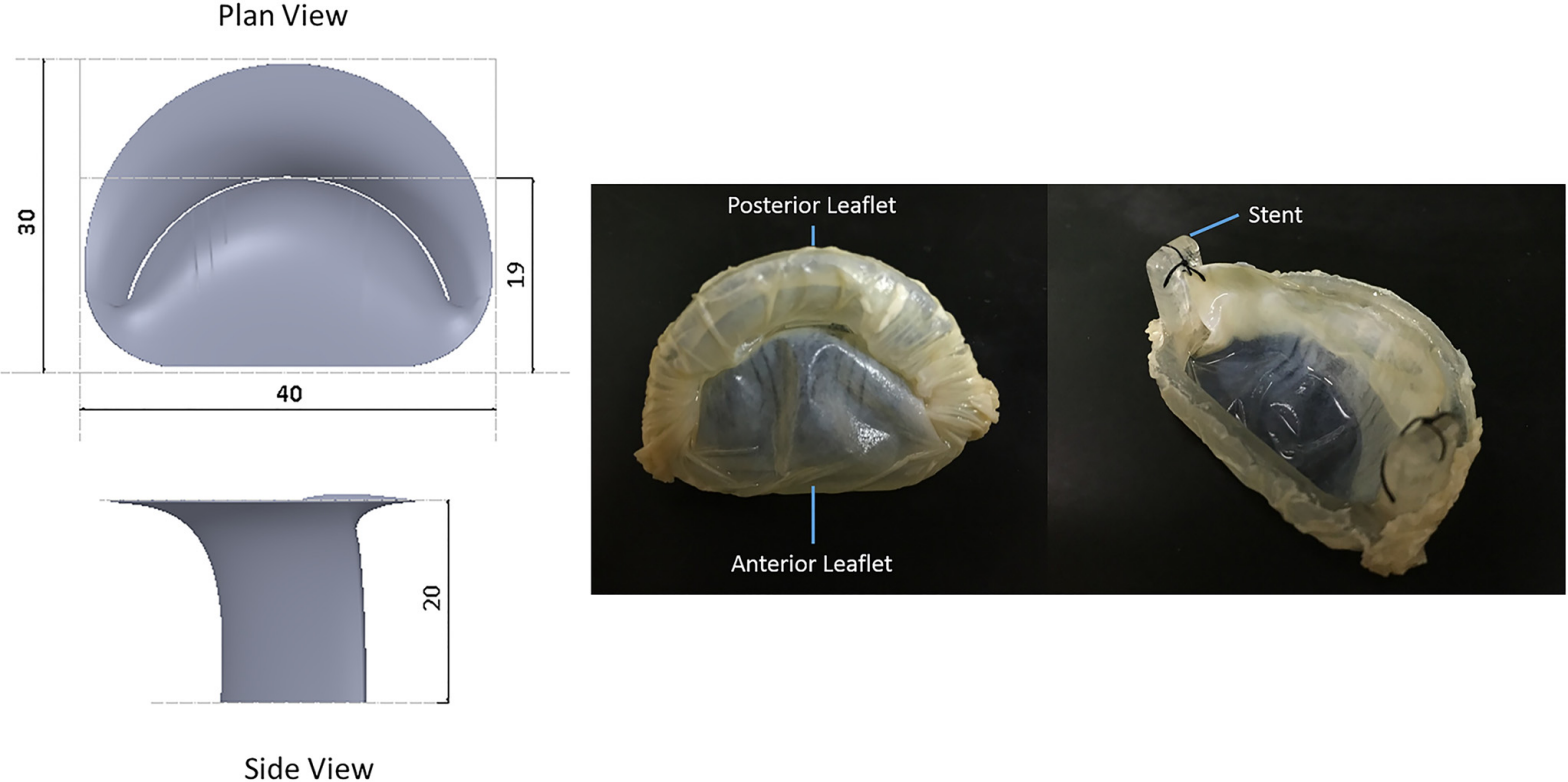
Engineering drawing of the bi-leaflet valve (GV valve) with the replica of the GD valve prototype. All dimensions in mm.

The well-established and clinically approved [16] tri-leaflet Epic Valve is employed in this study as a control in order to provide a standard at which the hemodynamic performance of the novel bi-leaflet bio-prosthesis can be compared (Fig 1). The Epic Valve has been shown to have satisfactory hemodynamic performance and durability at the 4^th^ year of implantation in a clinical study[16].

### D-shape bi-leaflet bio-prosthesis (GD Valve)

The D-shaped valve was (Fig 1) fabricated from porcine pericardium fixed in 0.625 percent glutaraldehyde solution[17] over-night and subsequently stored in approximately 0.2 percent glutaraldehyde solution at 4 degrees Celsius prior to testing.

The leaflet design was based on dimensions obtained from a study by Wang et. al.[18], consisting of one smaller posterior leaflet and a larger anterior leaflet (Fig 1). Upon fixation in glutaraldehyde, the pericardium was sutured onto a D-shaped stent fabricated by a 3D Objet260 printer (Stratasys, Eden Prairie, MN).

### Experimental Set-up

#### Left Heart in vitro simulator

**Fig 2:**
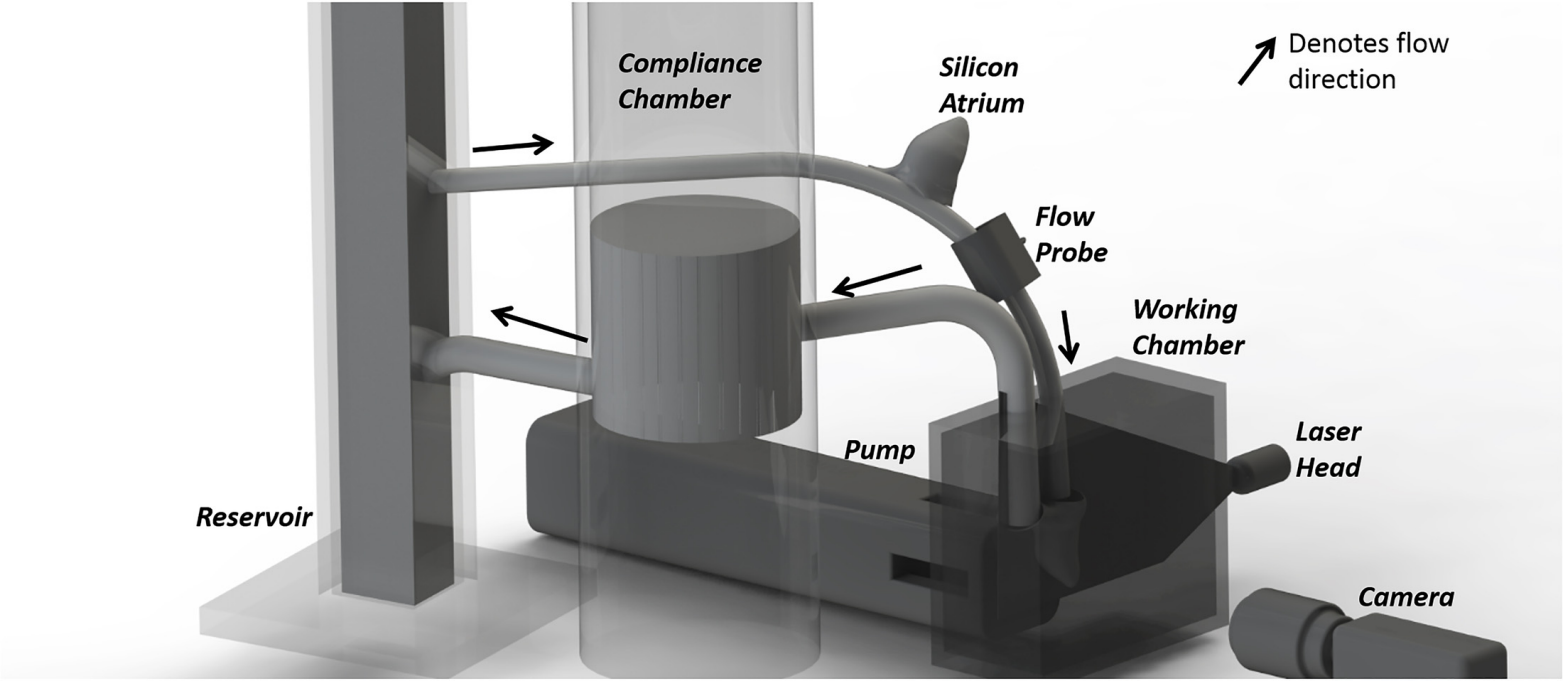
Schematic of experimental set up configured for particle image velocimetry. Arrows denote flow direction.

**Fig 3:**
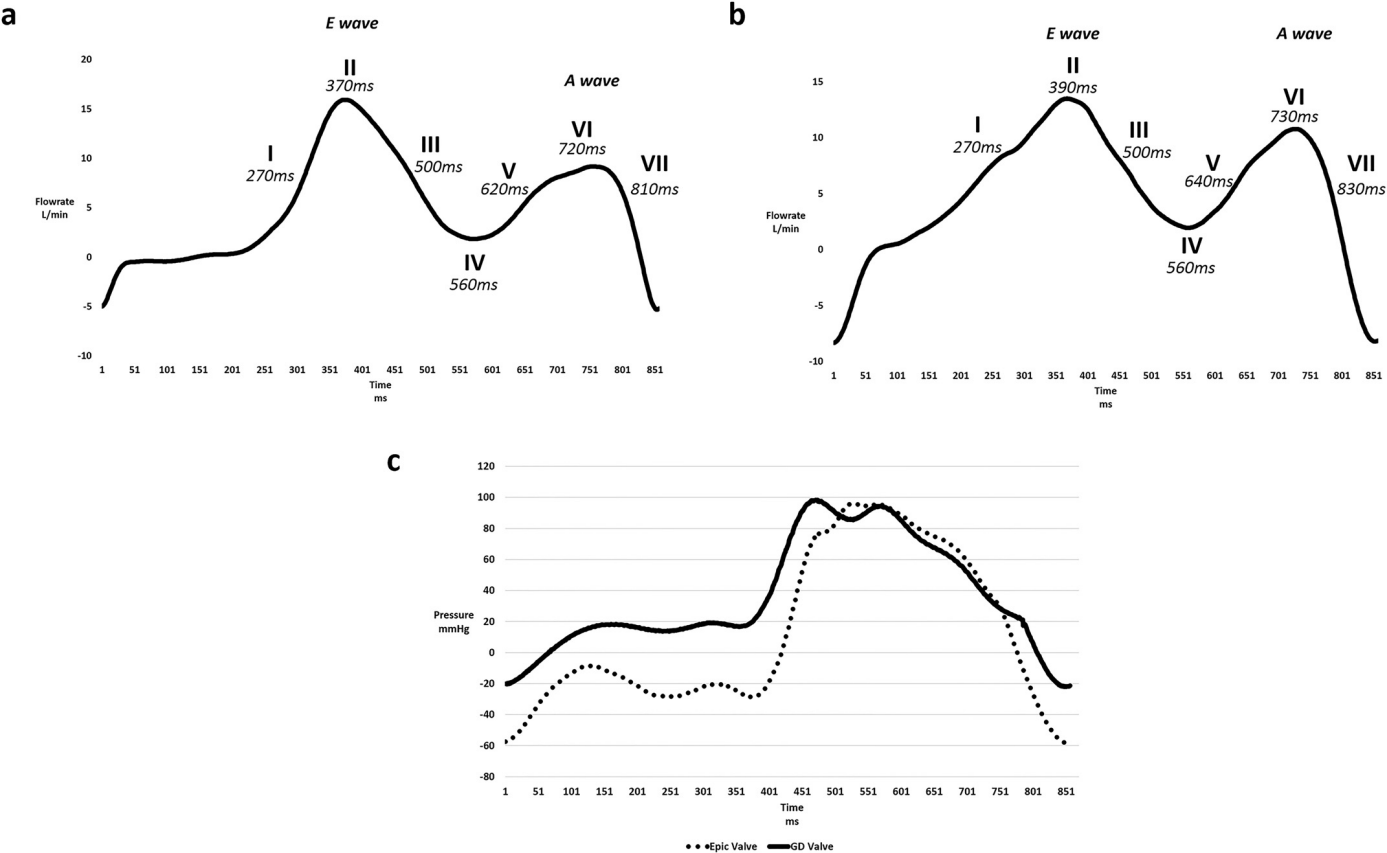
Samples of mitral waveforms observed. (a) A mitral flow waveform sample observed in the Epic Valve experiment. (b) A mitral flow waveform sample observed in the GD Valve experiment. (c) A LV pressure waveform sample observed in the Epic and GD valve experiment.

Blood analog of 40 percent glycerin in water (by volume) is circulated around the flow loop consisting of a working chamber, a reservoir and a compliance chamber by a pulsatile pump (ViVitro Labs Inc., Victoria, BC) (Fig 2). The system is calibrated with parameters summarized in Table 1, producing a physiological flow and pressure waveform (Fig 3). The generation of physiological trans-mitral flow waveform shown in Fig 3A and 3B, aided by a silicon atrium was measured by an ultrasound flow probe (Transonic Systems Inc., Ithaca, NY) attached immediately upstream of the mitral position. The flow probe was connected to a flow meter (Transonic Systems Inc., Ithaca, NY) calibrated to a 40% glycerin-water mixture during the PIV experiments.

**Table 1:** Summary of physiological parameters during PIV.

	Units	Epic Valve	GD Valve
**Heart Rate**	Beats/min	70	70
**Stroke Volume**	ml/Beat	86	80.1
**Average Flow Rate**	L/min	4.5–4.7	4.7–5.1
**Peak LV Pressure**	*mmHg*	95–107	98–104

#### Particle Image Velocimetry (PIV) Set-up

The flow loop was seeded with orange fluorescent 50–60μm particles with an approximate density of 1000 Kg/m^3^ (Cospheric LLC, Santa Barbara, CA). The synchronized charge coupled device (CCD) camera(Imager pro X, LaVision, Germany) was placed perpendicular to the ~1 mm thick laser sheet emitted by a Nd:YAG laser (Nano S PIV, Litron Lasers, UK) (Fig 2) which illuminated the mid-plane of the ventricle. An interrogation window size (with 6 to 12 particles) of 64 x64 pixels was found suitable to visualize the flow field. The velocity profile was obtained by cross-correlating a pair of consecutive images separated by a 30 to 50 μs time interval to ensure that the average particle displacement was less than 16 pixels. An average velocity profile was then obtained from 150 image pairs. The pulsatile pump, laser system and high speed camera are connected and synchronized for image acquisition at 7 time steps throughout the cardiac cycle (Fig 3A and 3B).

### Analysis

Post Processing analyses were done based on the PIV results obtained over 150 cycles (n = 150).

#### Reynolds decomposition

At each point in the flow field, the velocity was decomposed into a mean velocity component and a fluctuating velocity component as follows:

$$V_{i}=\;\bar{V}+\overset{´}{v}$$ (1)

Where *V*_*i*_ is the instantaneous velocity, $\bar{V}$ is the mean velocity over 150 cardiac cycles and $\overset{´}{v}$ is the turbulent velocity fluctuation.

#### Root-mean-square (RMS) of turbulent velocity fluctuation

RMS velocity was calculated as:

$$\bar{\overset{´}{v}}=\sqrt{\frac{1}{n}\sum_{i=1}^{n}\overset{´}{v}}$$ (2)

Where n = 150 cardiac cycles

#### Turbulence Intensity

Turbulence intensity which is a relation between the mean flow and the fluctuating velocity component, also referred to as the level of turbulence can be measured in percentage as:

$$I=\;\frac{\bar{\overset{´}{v}}}{\bar{V}}\times 100$$ (3)

Where $\bar{\overset{´}{v}}$ refers to the average of the root-mean-squared fluctuating components v_x_^`^, v_y_^`^and v_z_^`^:

$$\bar{\overset{´}{v}}=\;\sqrt{\frac{1}{3}\left(v_{x}^{`2}+v_{y}^{`2}+v_{z}^{`2}\right)}$$ (4)

Where $\bar{V}$ is computed from 3 mean velocity components V_x_, V_y_ and V_z_

$$\bar{V}=\;\sqrt{V_{x}^{2}+V_{y}^{2}+V_{z}^{2}}$$ (5)

#### Mean Kinetic Energy (MKE)

MKE was obtained by calculating the average kinetic energy from the mean velocity component at every spatial point in the left ventricle at each time step in the cardiac cycle:

$$\text{MKE}=\frac{1}{2}\rho {\left|\bar{V}\right|}^{2}$$ (6)

Where *ρ* is the density of the fluid taken to be 1100kg/m^3^

#### Turbulent Kinetic Energy (TKE)

TKE was estimated from the root-mean-square of the fluctuating velocity component approximates the intensity of the kinetic energy associated with turbulence:

$$\text{TKE}=\frac{1}{2}\rho {\left|v`\right|}^{2}$$ (7)

Where |v`| is the root-mean-square velocity magnitude of the fluctuating component.

#### Reynolds Shear Stress (RSS)

RSS was estimated from the root-mean-square of the fluctuating velocity component and it approximated the shearing stress exerted on the red blood cells and platelets due to turbulence.

$$\text{RSS}=\rho \bar{\overset{´}{u}}\;\bar{\overset{´}{v}}$$ (8)

Where $\bar{\overset{´}{u}}$ and $\bar{\overset{´}{v}}$ are the root-mean-square velocities fluctuating component.

#### Vorticity and Circulation

Vorticity: local rotational motion of a fluid at a particular spatial point and is computed as:

$$\omega =\;\nabla \;\times \;|\bar{V}|$$ (9)

Circulation: Integral of vorticity within a vortex area or interest and is computed as:

$$\Gamma =\;\int_{A}\omega .n\;dS=\omega_{A}.A$$ (10)

Where *ω* is the vorticity vector normal to the plane of interest, *ω*_*A*_ is the average vorticity within the bounded area and A is the area bounded by the curve S.

#### Vortex identification, λ2 criterion

The *λ*_*2*_ *criterion* by Jeong and Hussain (1995), enables vortex structures to be identified in the left ventricle flow field by separating the swirling motion of the fluid from the unsteady non rotational straining and shearing components in the flow[19]. The vortex structure is defined as the connected regions in the flow field where 2 eigenvalues of the tensor *S*^2^ + Ω^2^ are negative. Where *S* and Ω are the symmetric and anti-symmetric components of velocity gradient ∇**V:**

$$S=\;\frac{1}{2}\left(v_{i,j}+v_{j,i}\right)$$ (11)

$$\Omega =\;\frac{1}{2}\left(v_{i,j}-v_{j,i}\right)$$ (12)

Tensor *S*^2^ + Ω^2^ is symmetric thus it has only real eigenvalues which are ordered as *λ*_1_ > *λ*_2_ > *λ*_3_, thus vortex structure is defined as the area in the flow field where *λ*_2_ < 0.

#### Net Vorticity and Circulation

Net vorticity is defined as the vorticity within the areas of the vortex structures detected by the λ_2_ criterion. Likewise, net circulation is defined as the integral of the vorticity within vortex structures defined by the λ_2_ criterion.

## Results

This section analyzed the hemodynamics performance of both the St. Jude Epic valve and the D-shaped bi-leaflet valve. The valves were evaluated based on traditional hemodynamic parameters such as Reynolds Shear Stress (RSS), Turbulent Kinetic Energy (TKE) and Mean Kinetic Energy (MKE). In addition, the vortex formation in the LV downstream of each valve and its resulting circulation was analyzed and compared.

### Left ventricular velocity fields

**Fig 4:**
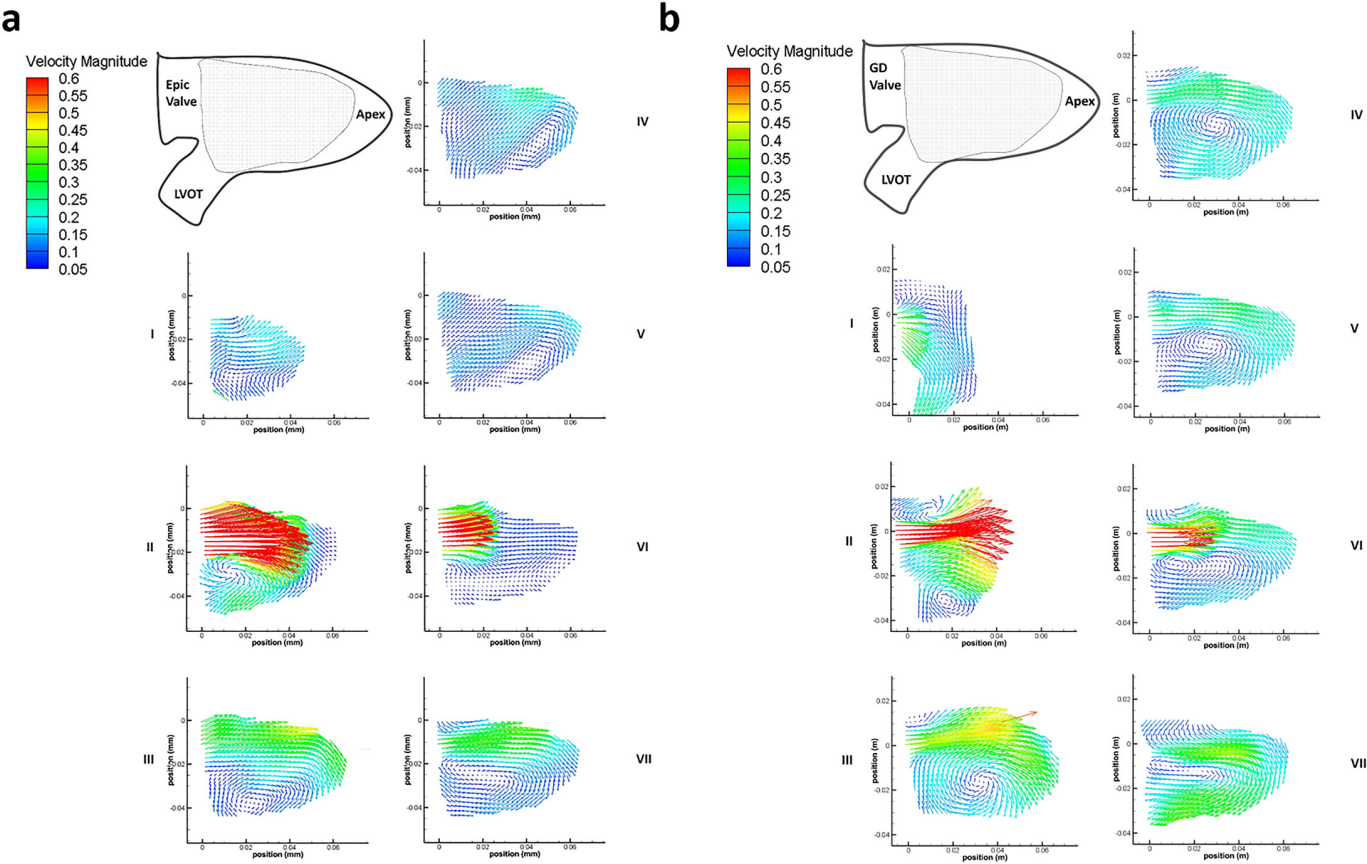
Velocity flow fields. (a) Velocity field [ms^-1^] of the Epic valve in the left ventricle over 7 time steps in 1 cardiac cycle. Shaded area denotes area of interest measured by PIV. (b) Velocity field [ms^-1^] of the GD bileaflet valve in the left ventricle over 7 time steps in 1 cardiac cycle. Shaded area denotes area of interest measured by PIV.

The side-by-side comparison of velocity vectors downstream for both Epic and GD valves for time steps I to VII of the cardiac cycle are shown in Fig 4A and 4B. At time step I, the flow accelerated pass the mitral valve as the left ventricle began to relax during diastole. Unlike that of the Epic valve where the flow was directed downwards towards the apex, the streamlines distal to the GD valve tended towards the left ventricular outflow track (LVOT). At the height of the E wave (Fig 3), the peak velocity of both the Epic and GD valves was observed at 1.42m/s and 0.96m/s, respectively (time step II). In both cases, vortex structures were formed in the LV due to adverse pressure gradients as the high velocity jets entered the lower flow region in the LV. Time Steps III, IV and V represent the pause between the initial E wave entry jet and the subsequent secondary jet due to left atrial contraction (A wave). It was during these 3 time steps (III, IV, V) that the streamlines of the two valves began to differ; at time step III of the GD valve, the vortex core was observed at the center of the ventricle and remained at the same location throughout all three time steps. This large centralize vortex can be seen redirecting the incoming fluid around the apex and towards the LVOT. However, in Epic valve, it was observed that the vortex core located near the LVOT migrated down towards the apex at time steps IV and V which then became elongated near the apex and subsequently directed the LV flow away from the LVOT towards the apex.

### Mean Kinetic Energy

**Fig 5:**
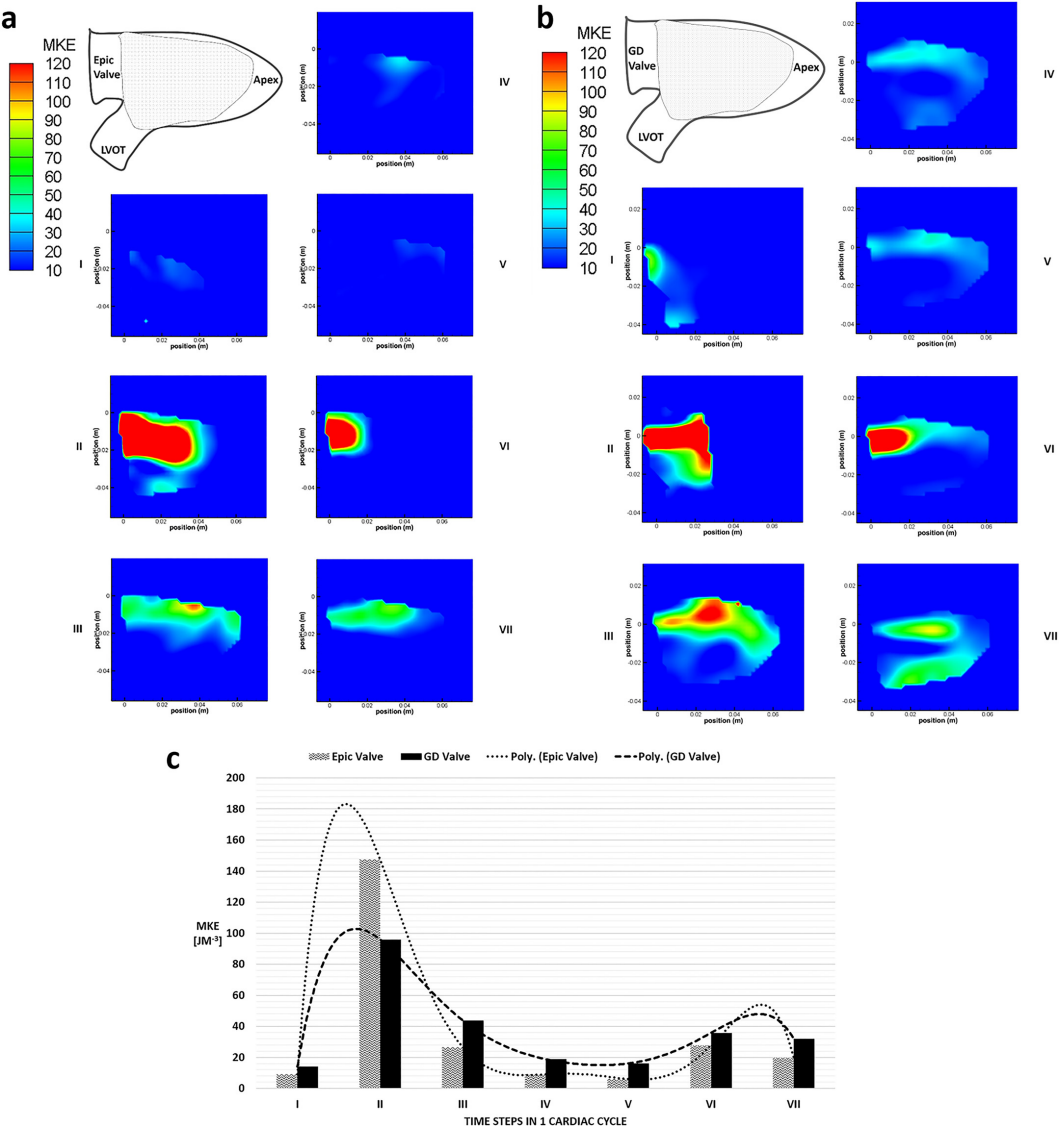
MKE [Jm^-3^] contour plots. (a) MKE [Jm^-3^] contour plot of the Epic valve in the left ventricle over 7 time steps in 1 cardiac cycle. Shaded area denotes area of interest measured by PIV. (b) MKE [Jm^-3^] contour plot of the GD valve in the left ventricle over 7 time steps in 1 cardiac cycle. Shaded area denotes area of interest measured by PIV. (c) A comparison of the average MKE [Jm^-3^] values in the left ventricle between EV and GD valves over 7 time points in 1 cardiac cycle. Poly—refers to fitted values by polyline.

Both valves exhibited high levels of MKE at the peak of the E and A waves at time steps II and VI (Fig 5A and 5B). The incoming jet from the Epic valve was seen to be larger (~26mm wide) and more centralized than that of the GD valve which was narrower (~17mm wide) and flowed along the contour of the posterior ventricular wall. Unlike the Epic valve, the MKE contours of the D-shaped valve suggested that more kinetic energy of the trans-mitral jet, observed at the apex of the LV, was conserved before the flow rotated towards the LVOT. These flow characteristics was similarly observed at time steps IV, V and VII for the GDV. Furthermore, the GD valve displayed a greater MKE of approximately 60 J/m^3^ near the LVOT region at time step VII (Fig 5B).

The average MKE was computed by taking the spatial averaging of the MKE in the mid-plane area of the LV and plotted over the 7 time steps in the cardiac cycle (Fig 5C). In the case of the Epic valve, the MKE within the LV peaks at the maximum of 147 J/m^3^, higher than that of the D-shaped GD valve at 96 J/m^3^ (Time step II). However, after the peak flow at time step II, MKE values for the Epic valve throughout the rest of the cardiac cycle were lower than that of the D-shaped GD valve.

### Vorticity and Circulation

**Fig 6:**
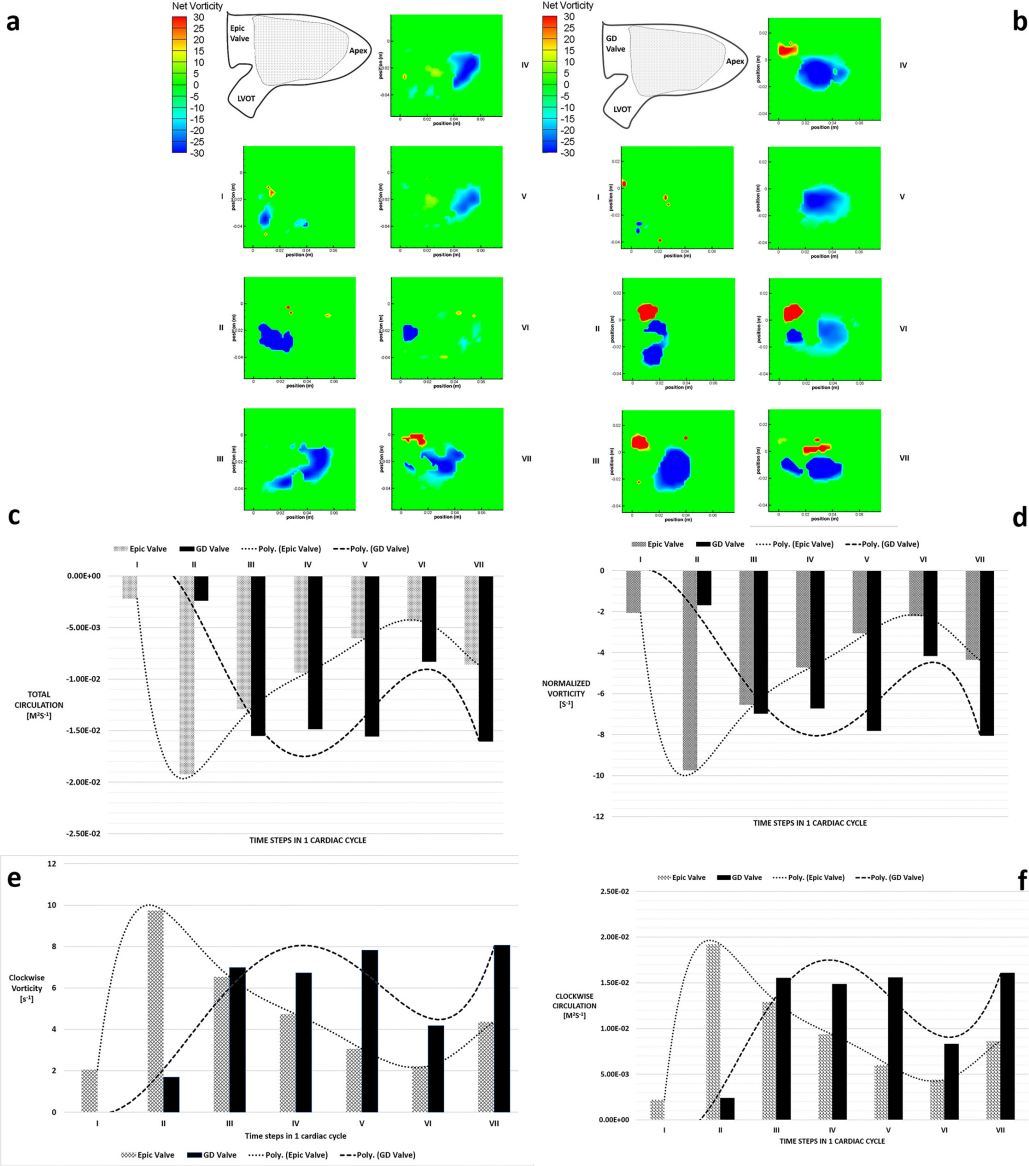
Vorticity and Circulation plots. (a) Net vorticity [s^-1^] contour plot of the Epic valve in the left ventricle over 7 time steps in 1 cardiac cycle. Shaded area denotes area of interest measured by PIV. (b) Net vorticity [s^-1^] contour plot of the bileaflet GD valve in the left ventricle over 7 time steps in 1 cardiac cycle. Shaded area denotes area of interest measured by PIV. (c) Total circulation in the left ventricle for both valves over 7 time steps in 1 cardiac cycle. Poly—refers to fitted values by polyline. (d) Normalized vorticity in the left ventricle for both valves over 7 time steps in 1 cardiac cycle. Poly—refers to fitted values by polyline. (e) Clockwise vorticity in the left ventricle for both valves over 7 time steps in 1 cardiac cycle. Poly—refers to fitted values by polyline. (f) Clockwise circulation in the left ventricle for both valves over 7 time steps in 1 cardiac cycle. Poly—refers to fitted values by polyline.

Left ventricular vortex structures for both cases were identified via the λ_2_ criterion with the aim of isolating the rotational components of the vortex. Fig 6A and 6B show the development of vortex structures for both valves over 1 cardiac cycle over 7 time steps. At peak flow (E wave) for the case of the D-shaped GD valve, a pair of counter-rotating vortices were observed at the wake of the incoming jet at time step II (Fig 6B), whereas only a single clockwise vortex core was observed downstream of the Epic valve. At this stage, the trans-mitral jet of the Epic valve led to higher overall circulation of magnitude ~1.92E^-2^ m^2^s^-1^ compared to the GD valve (~-2.39E^-3^ mm^2^s^-1^) (Fig 6C). It was observed in the GD valve that the overall LV circulation was negative (clockwise) for time steps II to VII as the clockwise vortex structure was seen to be larger than that of the counterclockwise one. The net vorticity contours of the GD valve showed a large circular region of negative vorticity at the center of the LV throughout the cardiac cycle after the initial filling jet. This area of negative vorticity reached a maximum size of approximately 30mm x 40mm, covering nearly the entire LV especially during the time steps IV and V, corresponding to the time interval between the initial E wave and the A wave. In contrast, the negative vortex regions found in the LV with the Epic valve were irregular and was typically observed to moved towards the apex after trans-mitral jet entered the LV at both peak flows (E wave and A wave). Furthermore, it is observed that the averaged normalized LV vorticity at time steps III to VII is found to have a greater magnitude in the case of the bileaflet GD valve as compared to the Epic valve (Fig 6D).

Clockwise circulation is vital as it diverts the incoming filling jet around the apex and towards the LVOT and aorta[8]. Total clockwise circulation and vorticity for each time point was computed for both valves over one cardiac cycle (Fig 6E and 6F). The Epic valve’s clockwise circulation peaks at time step II (1.92E^-2^ m^2^s^-1^) where the filling jet entered the LV, and then declined over time to a minimal level of 4.39E^-3^ m^2^s^-1^ before rising slightly at time step VII (Fig 6F). Unlike the significant decline observed in the Epic valve, the clockwise circulation observed in the D-shaped GD valve increased exponentially reaching a peak of 1.55E^-2^ m^2^s^-1^ (Time step III) before plateauing at approximately ~1.5E^-2^ m^2^s^-1^ at time steps IV, V and VII, except at the A wave where the circulation drops to ~8.31E^-3^ m^2^s^-1^. In addition, after the initial filling jet (E wave), the overall circulation in the LV for the GD valve was more negative than that seen in the Epic valve (Fig 6C), indicating that in spite of the presence of a positive vortex core (Fig 6B), the swirl of the streamlines was still strongly directed towards the LVOT.

### Reynolds Shear Stress (RSS), Turbulent Kinetic Energy (TKE) and Turbulence Intensity (TI)

**Fig 7:**
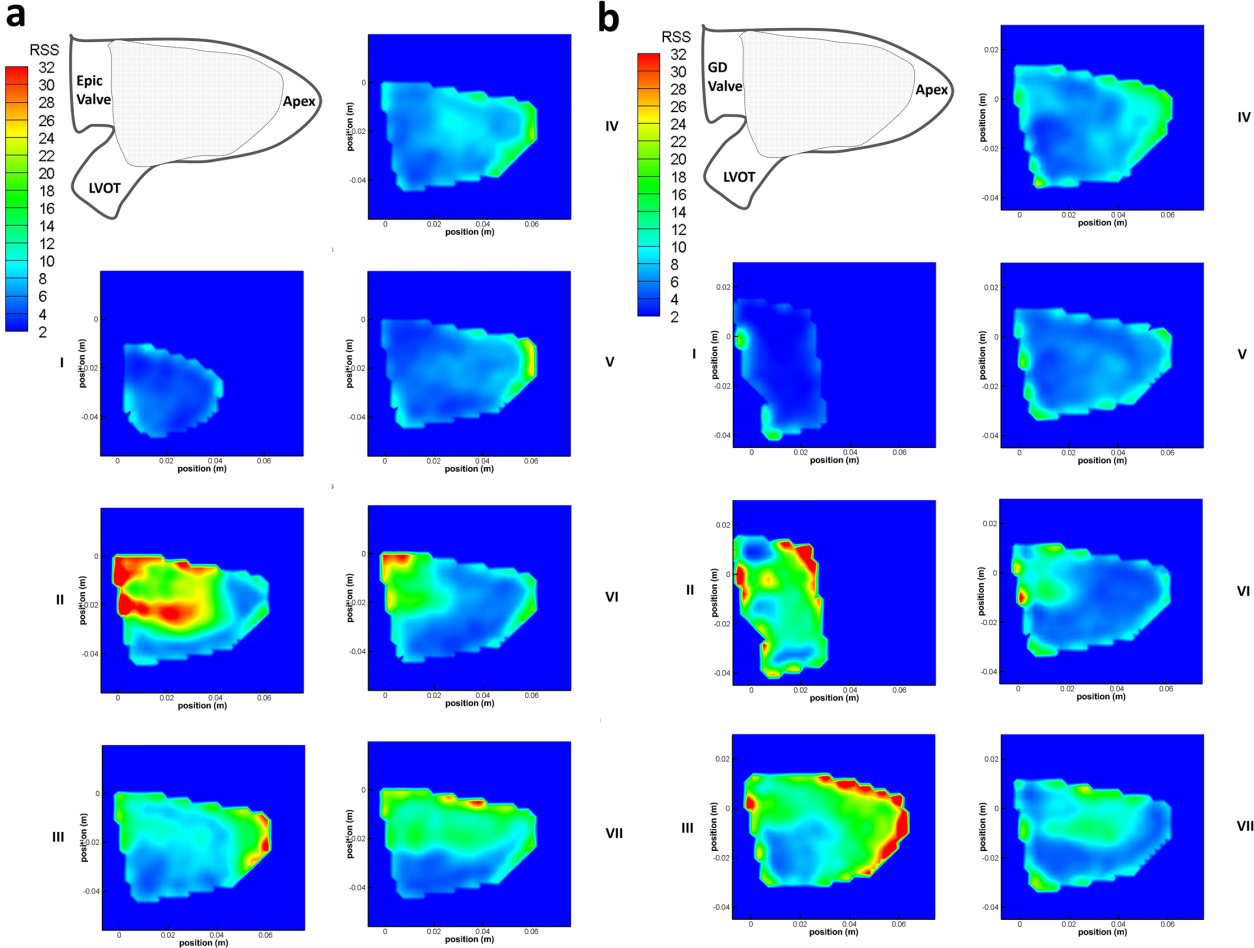
RSS [Pa] contour plots. (a) RSS [Pa] contour plot of the Epic valve in the left ventricle over 7 time steps in 1 cardiac cycle. Shaded area denotes area of interest measured by PIV. (b) RSS [Pa] contour plot of the bileaflet GD valve in the left ventricle over 7 time steps in 1 cardiac cycle. Shaded area denotes area of interest measured by PIV.

**Fig 8:**
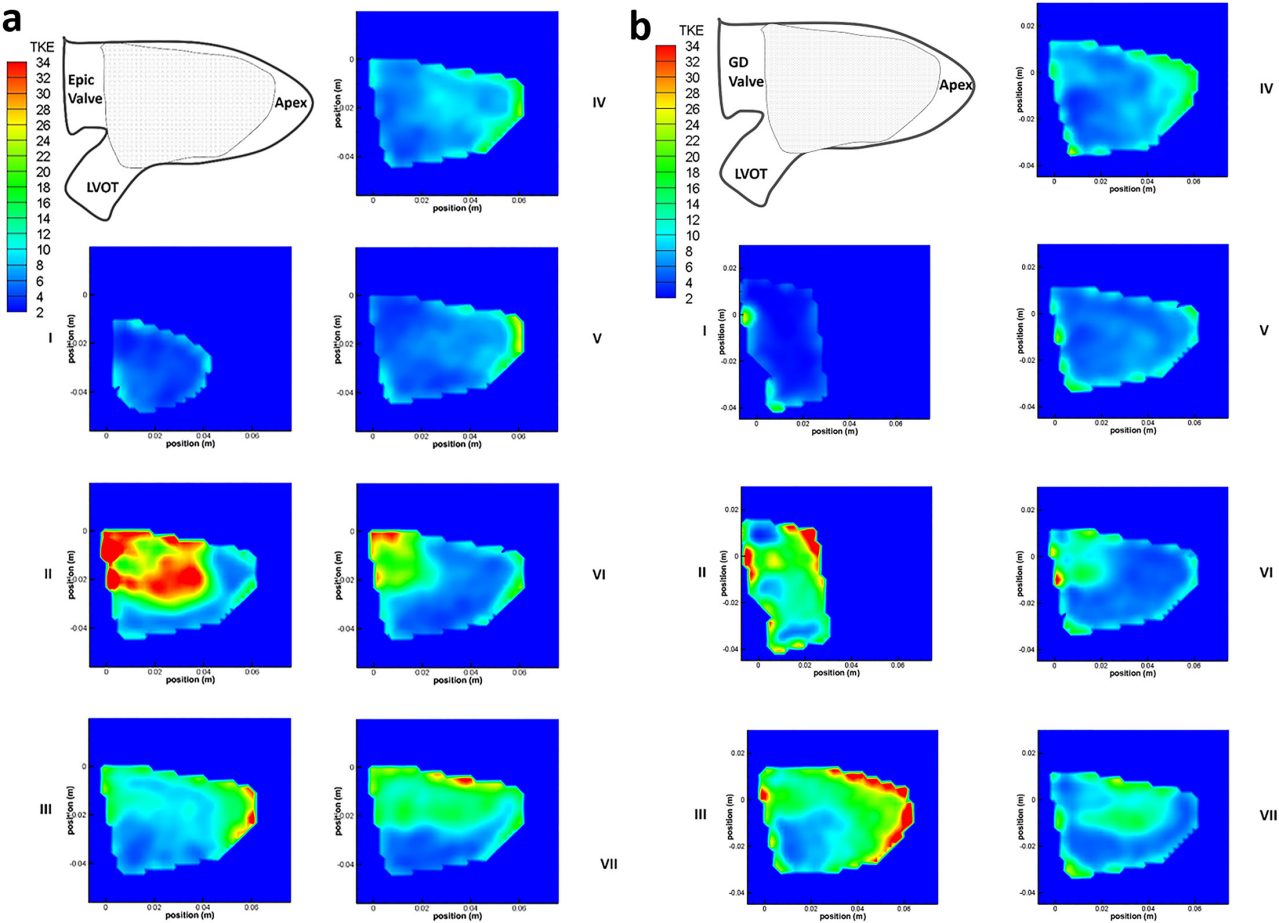
TKE [m^2^s^-2^] contour plots. (a) TKE [m^2^s^-2^] contour plot of the Epic valve in the left ventricle over 7 time steps in 1 cardiac cycle. Shaded area denotes area of interest measured by PIV. (b) TKE [m^2^s^-2^] contour plot of the bileaflet GD valve in the left ventricle over 7 time steps in 1 cardiac cycle. Shaded area denotes area of interest measured by PIV.

The maximum RSS level produced by both valves were ~60Pa at time step II (Fig 7A and 7B) which is lower than the reported threshold level of 400Pa for hemolysis to occur[20]. The maximum TKE for both Epic and GD valves occurred at time step II (Fig 8A and 8B), where the flow was turbulent with a peak value of 112 J/m^3^ and 62 J/m^3^ respectively.

**Fig 9:**
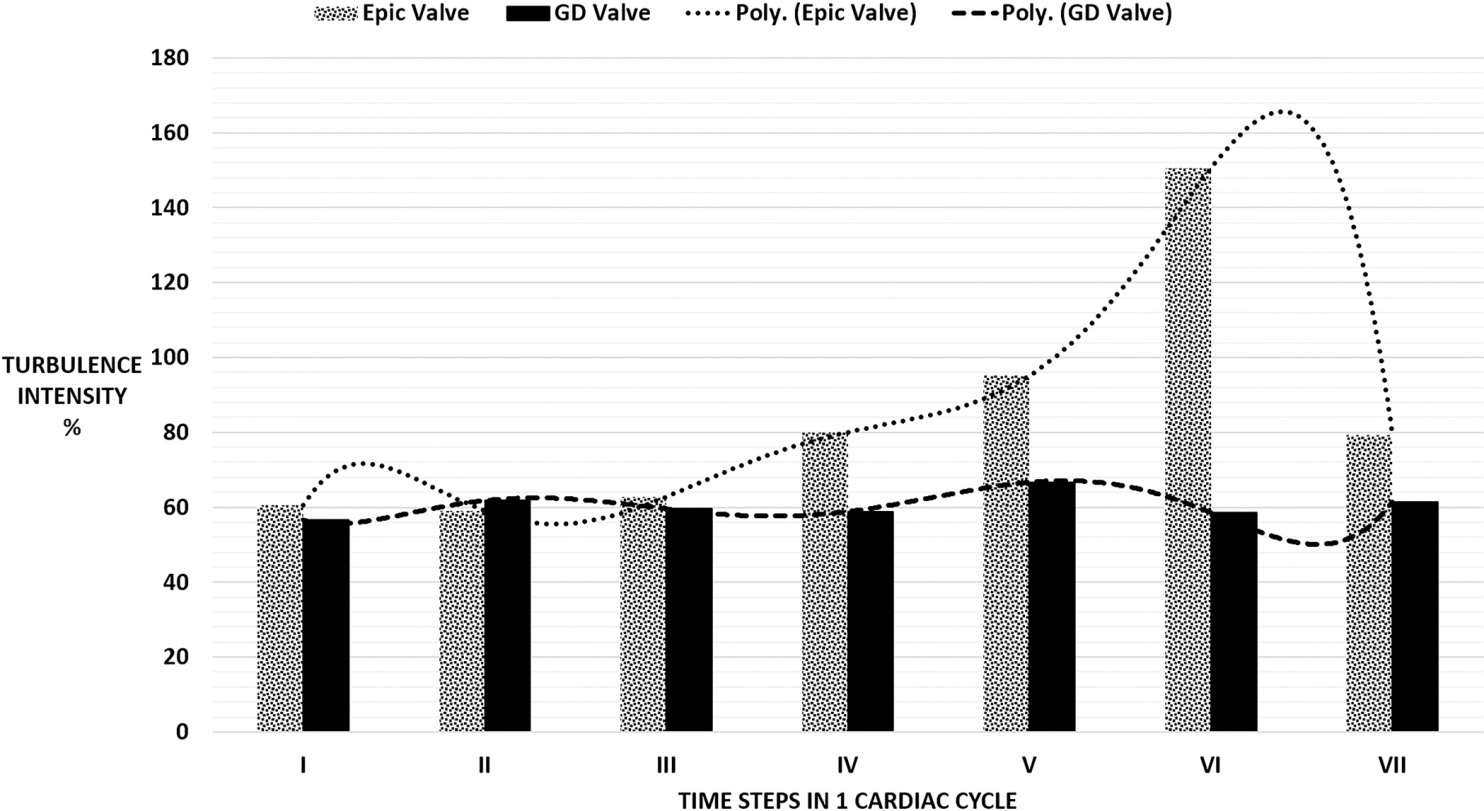
Left ventricular turbulence intensity for both valves over 7 time steps in 1 cardiac cycle. Poly—refers to fitted values by polyline.

Although the maximum RSS values for both valves were not significantly different, the TKE values computed for the Epic valve was 1.8 times greater than that of the D-shaped valve. Furthermore, the turbulence intensity of the case of the Epic valve was higher than that of the D-shaped GD valve at every time step in the cardiac cycle, especially at time steps IV to VII (Fig 9). The peak turbulent intensity for the Epic valve occurred at time step VI (151 percent) where the second filling jet entered the LV during the A wave.

## Discussion

Prior *in vivo* studies involving imaging techniques have established the fact that in a healthy heart, there exists a large asymmetric clockwise vortex located at the center of the LV during ventricular diastole [4, 8, 21–23]. This asymmetric clockwise vortex which fills nearly the entire LV during the interval between diastole and systole conserves kinetic energy of the blood as it is redirected from the mitral valve to the aorta [4]. Many studies have shown that artificial heart valves do not replicate physiological flow field, leading to higher turbulence levels in the LV [24]. This study showed the first time in an in-vitro set up similar to the design employed by Okafor et al.[25], a D-shaped artificial valve, as in the native valve configuration, is able to both preserve asymmetric clockwise vortex and conserve kinetic energy during ventricular diastole.

### Velocity field and vortex formation

#### Epic Valve

The velocity fields of the two heart valves start to differ significantly at time step IV during the mid-diastolic diastasis between the E wave and the A wave (Fig 4A and 4B). In the D-shaped GD valve, the circulation around the center core resulted in a higher mean kinetic energy near the anterior of the LV of approximately 10–20 J/m^3^, directing the flow towards the LVOT. In contrast, in the case of the Epic valve, flow was directed away from the LVOT with a velocity of ~0.16–0.06 m/s (time step IV and V). Furthermore, the significantly lower mean velocity magnitude observed in the anterior region of the LV (time steps VI and VII, Epic valve) could be due to the vector canceling effect because of directional differences between the downward jet (A wave) and the direction of the circulating fluid in the region immediately distal of the mitral position. This was unlike that of the D-shaped GD valve where the direction of the A wave jet and the circulating fluid were similar in direction (towards the apex). This explains why the turbulence intensity level of the Epic valve in time step VI was more than double that of the GD valve (Fig 9) as a drop in mean flow and an increase in flow disturbances and instability could result in a higher turbulent intensity and greater energy dissipation due to a higher peak TKE level.

#### D-shaped GD Valve

The LV velocity flow field of the D-shaped GD valve (Fig 4B) during diastole was similar to that seen in a healthy heart in-vivo [8, 22, 26], where the incoming filling jets (E wave and A wave) merged the existing circulating fluid in the LV smoothly before redirected towards the LVOT efficiently. With the λ_2_ criterion method of vortex core detection, we were able to identify a large clockwise vortex that nearly filled the entire LV (Fig 6B) during time steps III, IV and V, similar to that in a healthy heart [10, 13]. This large clockwise vortex maintained throughout the diastolic period after its formation at time step II where the initial filling jet (E wave) entered the LV (Fig 6B), is the reason for the clockwise LV circulation observed in the D-shaped GD valve and not in the Epic valve. This vortex was sustained at an approximately constant level of ~7–8 s^-1^ at time steps III, IV, V and VII (Fig 6E). We postulate that it is this negative (clockwise) LV circulation that aided in the redirection of the blood to the LVOT thus reducing energy loss (Fig 5B) and turbulence (Fig 9).

#### Energy conservation

At time step II, the initial filling jet of the Epic valve was higher in Mean Kinetic Energy (MKE) when compared to that of the GD valve (Fig 5C), as more kinetic energy enters the system. However, this initial energy was significantly dissipated in subsequent time steps as observed in the lower levels of MKE of the Epic valve after time step II. We postulate that the large clockwise vortex seen in the D-shaped GD valve case previously described may play an important role in the maintenance of MKE levels (Fig 5C) in the LV especially during the interval between the two incoming jets, as prior studies have demonstrated the effect of vortex formation on energy dissipation[27]. Furthermore, the greater LV clockwise circulation in the D-shaped GD valve resulted in higher MKE levels in the region near the LVOT (Fig 5B). We hypothesize that the higher level of MKE at time step VII just before systole will result in better cardiac efficiency.

#### Turbulence

Prior studies have shown that RSS levels are closely related to hemolysis and platelet activation, with a threshold of approximately 400 Pa and 100 Pa respectively [28]. On the other hand TKE is associated with kinetic energy being dissipated by viscous stresses at the Kolmogorov microscales through the energy cascade model[29].

Although both the valves have approximately the same maximum RSS values, they differ in terms of turbulence intensity (TI). The TI of the D-shaped GD valve was observed to be significantly lower at time steps IV, V and VI as the mean velocity component is significantly higher than the fluctuating one. We hypothesize that in the case of the D-shaped GD valve, the large central vortex reduces velocity fluctuation while maintaining a high mean flow.

## Conclusion

Physiological flow patterns can be replicated by the biomimicry of a native mitral valve’s geometry. Physiological flow pattern in the LV is made up of a large central asymmetric vortex that enhances clockwise circulation towards the LVOT. This ensures stability and minimizes fluctuations in the flow while enhancing the conservation of mean kinetic energy, potentially leading to higher cardiac efficiency and lower turbulence. In addition, we suggest that at the mitral position, traditional hemodynamics performance parameters are insufficient to evaluate valve performance without taking into account LV vortex formation.

## Limitations

Since two dimensional PIV was employed to capture only the mid plane velocity field, the three dimensional nature of LV flow[30] was not fully reflected in this study. Furthermore, the data sets presented in this study represents an n = 1 as a single valve of each design was utilized.

## Supporting Information

S1 File Supporting Information. (PDF)
