# Supplementary material for: A D-Shaped Bileaflet Bioprosthesis which Replicates Physiological Left Ventricular Flow Patterns
Source: PLoS One. 2016 Jun 3;11(6):e0156580. doi: 10.1371/journal.pone.0156580 (PMC4892640; doi:10.1371/journal.pone.0156580)
Supplement: S1 File — (PDF) [file pone.0156580.s001.pdf]

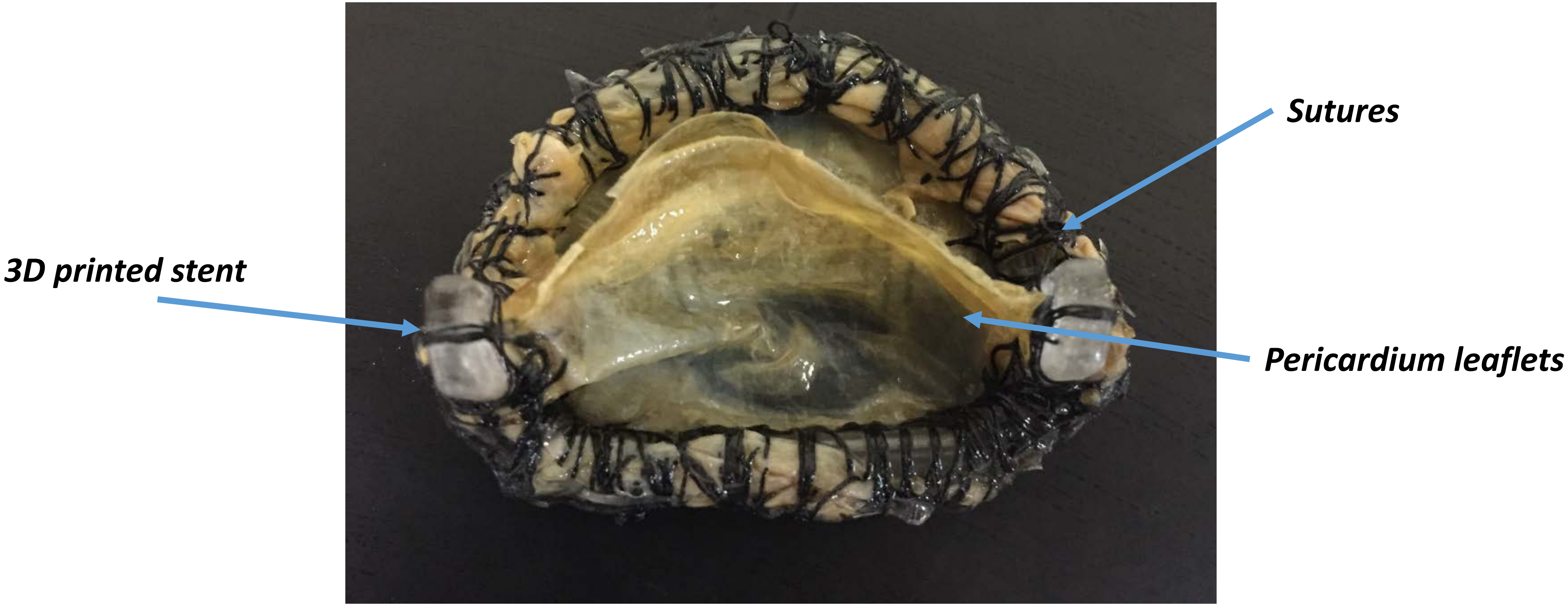

Figure 1: Bottom view of actual bileaflet valve employed in experiments.

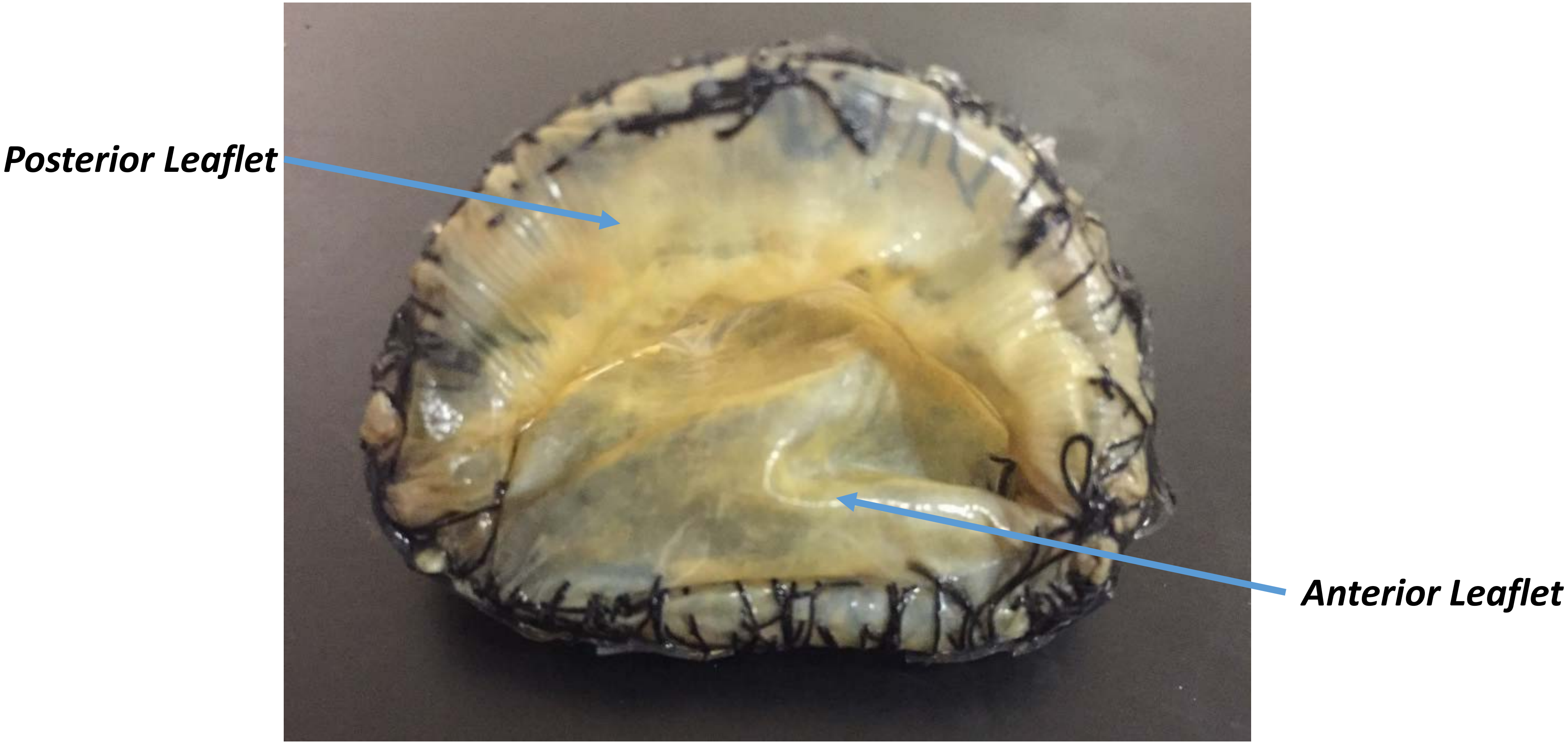

Figure 2: Top view of actual bileaflet valve employed in experiments.

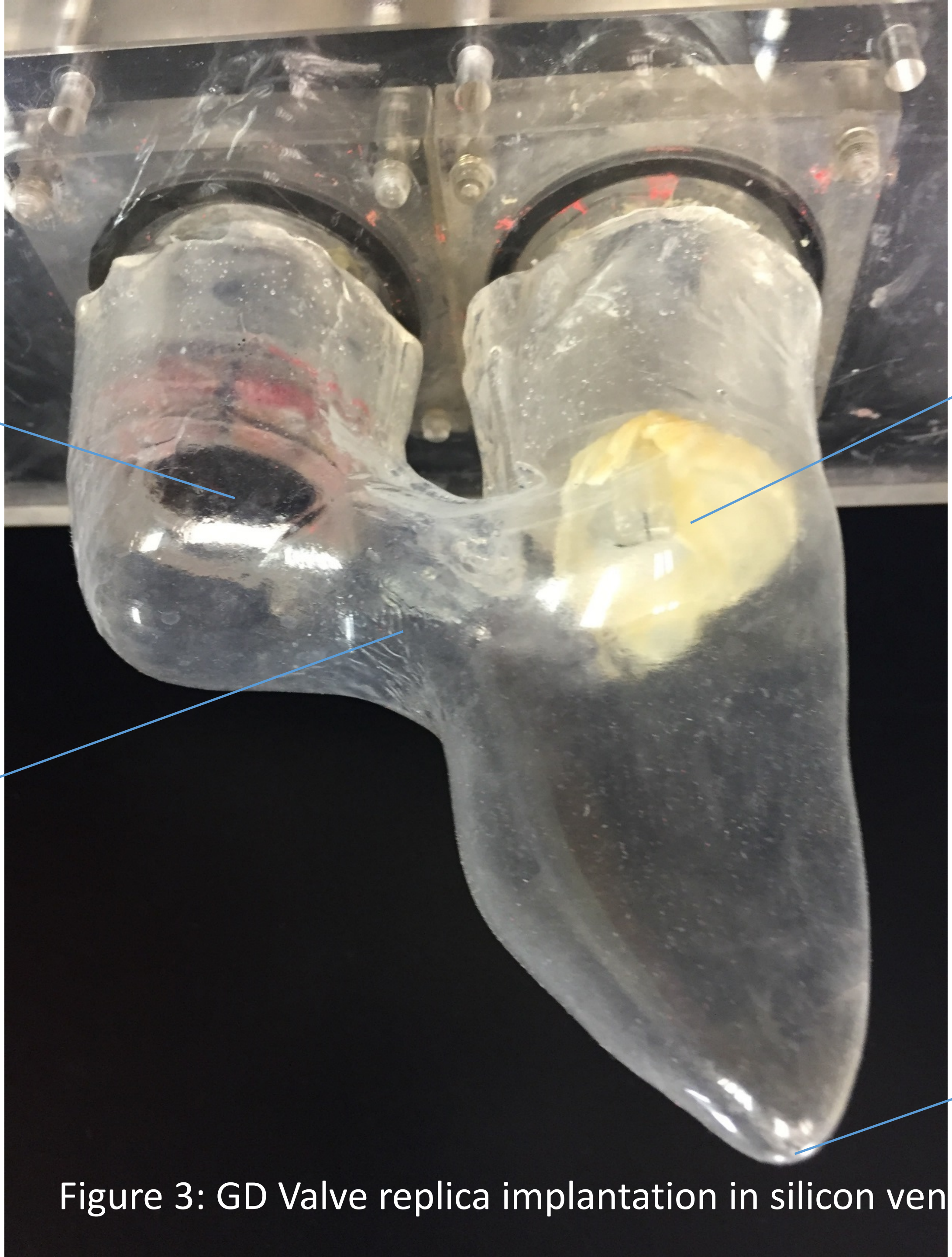

Figure 3: GD Valve replica implantation in silicon ven

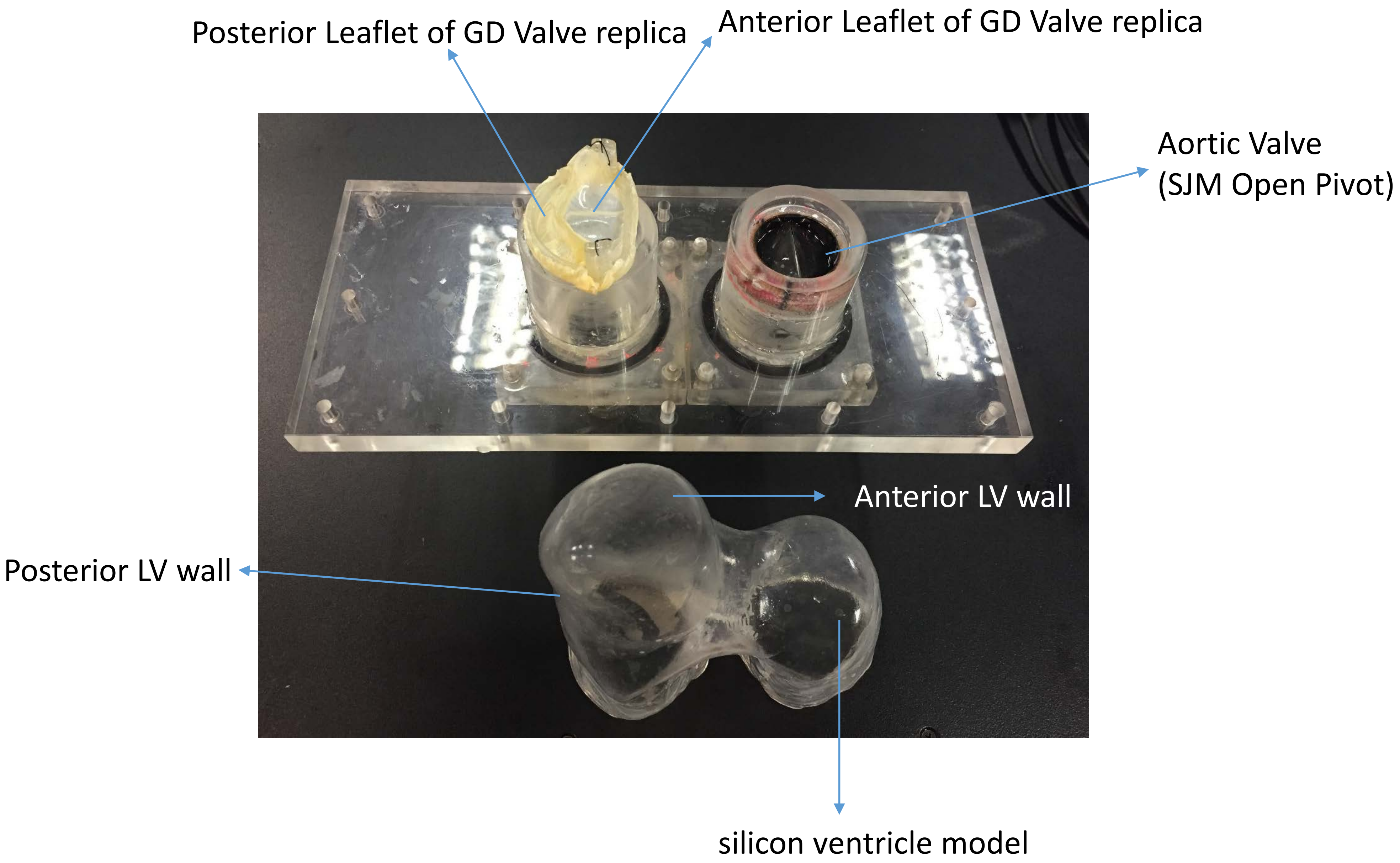

Figure 4: GD Valve replica implantation in silicon ventricle.
